# Physical, social, and psychological characteristics of community-dwelling elderly Japanese dog and cat owners

**DOI:** 10.1371/journal.pone.0206399

**Published:** 2018-11-14

**Authors:** Yu Taniguchi, Satoshi Seino, Mariko Nishi, Yui Tomine, Izumi Tanaka, Yuri Yokoyama, Hidenori Amano, Akihiko Kitamura, Shoji Shinkai

**Affiliations:** Research Team for Social Participation and Community Health, Tokyo Metropolitan Institute of Gerontology, Tokyo, Japan; Ehime University Graduate School of Medicine, JAPAN

## Abstract

**Objectives:**

Previous studies examined the physical characteristics of older dog owners. However, associations of health-related factors with dog/cat ownership have not been comprehensively evaluated. This cross-sectional study examined physical function, physical activity, social function, and psychological function of a population of community-dwelling older Japanese dog and cat owners after controlling for important confounders.

**Methods:**

The analysis included data from 11,233 community-dwelling adults aged 65 years or older (51.5% women; 52.3% aged 75–84), in Ota City, Tokyo, Japan. Pet ownership experience and pet species owned were determined by self-reported questionnaire, and current, past, and never dog/cat ownership was analyzed.

**Results:**

Analysis of variables related to physical function and physical activity showed that motor fitness scale and walking activity were significantly associated with experience of dog ownership, after adjustment for important sociodemographic and health characteristics. Analysis of social function showed that interaction with neighbors, social isolation, and trust in neighbors were significantly associated with experience of dog ownership and cat ownership.

**Conclusions:**

As compared with respondents with no history of pet ownership, motor fitness and walking activity are greater for dog owners and social function is higher for dog and cat owners. Caring for a dog or cat might be an effective health promotion strategy to increase physical activity and facilitate social participation among older adults.

## Introduction

The mutually beneficial and dynamic relationship between people and animals has been referred to as human–animal interaction, and accumulating evidence highlights the psychological [1], physiological, and social benefits of interaction with animals and the therapeutic potential of animal-assisted programs in a wide range of settings [2]. Previous studies reported the physiological benefits of such interaction among children and adults [3–10].

Nagasawa et al. reported that childhood experience of dog ownership was related to sociality in old age [11]. The effects of dog/cat ownership might accumulate over the owner’s life, and the benefits of a history of dog/cat ownership might be greater in later life than during youth. Thorpe et al. examined 2531 community-dwelling adults aged 71–82 years and reported that dog walkers at baseline were 1.65 times as likely as non–dog owners who did not walk at least three times per week to meet recommended walking targets within 3 years [12]. Dall et al. examined 43 pairs of dog owners and non–dog owners aged 65 years or older and reported that the dog owners had longer walking times and fewer sedentary events [13]. Although previous studies examined the physical characteristics of older dog owners, they did not assess social and psychological factors in this population or independent associations of health characteristics with dog/cat ownership after adjustment for sociodemographic characteristics. Dog/cat owners may have many opportunities to facilitate social participation and/or to maintain psychological health through higher physical activity.

This cross-sectional study examined sociodemographic and comprehensive health characteristics in a large sample of community-dwelling older dog/cat owners in Japan. In addition, we examined health characteristics such as physical function, physical activity, social function, and psychological function among dog/cat owners, after controlling for important sociodemographic characteristics. A comprehensive analysis of the effects of dog/cat ownership on health-related factors among older adults might yield insights regarding health promotion strategies in super-aging societies.

## Methods

### Participants

Data for this study were collected as part of a community-wide intervention trial (Ota Genki Senior Project) in Ota City, Tokyo in 2016. Ota City has 18 administrative districts, and the total population in 2016 was 716,645 (162,443 residents aged ≥65 years). We mailed a self-administered questionnaire to 15,500 older residents, and a total of 11,925 questionnaires were returned (76.9%). The details of the study design have been previously reported [14]. The present study was approved by the Ethical Committee of the Tokyo Metropolitan Institute of Gerontology (approved June 1 2016 and June 9 2017). To be eligible for the present study, individuals had to complete the questionnaire on experience of dog/cat ownership. Ultimately, data from 11,233 (rate of valid responses, 72.5%) community-dwelling adults aged 65 years or older were included in the analysis.

### Definition of dog/cat ownership

Participants were asked if they had lived with a pet (current, past, or never). Those with current or past pet experience were asked about pet species in the household (dog, cat, or other). These responses were used to classify dog ownership and cat ownership as current, past, or never.

### Other variables

The covariates included sociodemographic and characteristics (sex, age, living alone, household size, marital status, educational attainment, equivalent income, employment, history of chronic diseases, chronic pain, history of hospitalization during the past year, fall during the past year, alcohol drinking, smoking status, sleep duration, food variety, and Tokyo Metropolitan Institute of Gerontology Index of Competence [TMIG-IC] score), physical function and physical activity (mobility limitation, body mass index, Motor Fitness Scale, physical activity, and frailty status), social function (interaction with neighbors, social isolation, trust in neighbors, and frequency of going outdoors), and psychological function (subjective happiness, self-rated health, Geriatric Depression Scale [GDS]-5 score, and World Health Organization Five [WHO-5] Well-Being Index [15]).

The chronic diseases evaluated included clinically relevant medical conditions, namely, hypertension, hyperlipidemia, heart disease, stroke, diabetes mellitus, bone and joint disease, lung respiratory disease, and cancer. For each of these conditions, participants were asked if they had received a physician diagnosis (yes or no) [16–18]. Food variety was assessed by dietary variety score, which was calculated by using the consumption frequencies for 10 food items (meat, fish/shellfish, eggs, milk, soybean products, green/yellow vegetables, potatoes, fruit, seaweed, and fats/oils) during the week. The score ranges from 0 to 10, and higher scores indicate greater food variety [19]. The TMIG-IC is designed to measure higher-level competence in older community residents. The score ranges from 0 to 13, and lower scores indicate lower functional capacity [20]. Mobility limitation was defined as self-reported difficulty in walking one-quarter of a mile or climbing 10 steps without resting [21,22]. The Motor Fitness Scale was evaluated by using 14 items on basic motor ability. The score ranged from 0 to 14, and higher scores indicated greater motor ability [23]. Physical activity was assessed by using the International Physical Activity Questionnaires-Short Form. Moderate-to-vigorous and moderate physical activity and walking activity (metabolic equivalent [MET]-hours/week) were evaluated [24,25]. Frailty status was assessed by a modified version of the Kaigo-Yobo Checklist. The score ranges from 0 to 15, and a score higher than 4 was defined as frailty [26]. Interaction with neighbors was classified as close relationship, conversation level, exchange of greetings only, and no social contact. Social isolation was assessed by determining the frequencies of face-to-face and non–face-to-face contact with non-resident children, relatives and friends, or neighbors. An overall frequency of contact with others less than once a week was categorized as social isolation [27]. The WHO-5 is designed to measure psychological health. The WHO-5 score ranges from 0 to 100, and lower scores indicate lower positive mood and vitality.

### Statistical analyses

First, associations of sociodemographic and comprehensive health characteristics with current, past, or never dog/cat ownership were tested by using sex- and age-adjusted univariate cumulative logit models. Second, to examine independent associations of health characteristics with dog/cat ownership after controlling for potential confounders, we used mixed-effects cumulative logistic regression models and the SAS PROC GLIMMIX. The random effects were the study area. Potential confounders were sociodemographic and health characteristics significantly associated with dog/cat ownership in cumulative logistic regression (eg, sex, age, household size, living alone, educational attainment, equivalent income, history of cancer, hospitalization during the past year, fall during the past year, alcohol drinking status, TMIG-IC score). Living alone, marital status, employment, and smoking status were excluded because of collinearity (R>0.5). In the mixed-effects cumulative logistic regression models, we analyzed associations of physical function and physical activity, social function, and psychological function with dog/cat ownership. Statistical analyses were done with SPSS (version 18.0; SPSS, Inc., Chicago, IL, USA) or SAS (version 9.4; SAS Institute, Inc., Cary, NC, USA). A P value of less than .05 was considered to indicate statistical significance.

## Results

Overall, 6377 (56.8%) were never owners, 3311 (29.5%) were past owners, and 1545 (13.8%) participants were current dog/cat owners. Among 4856 current or past dog/cat owners, 2773 (57.1%) had owned a dog only, 1286 (26.5%) had owned a cat only, 498 (10.3%) had owned a dog and cat, 135 (2.8%) had owned a dog and another animal, 97 (2.0%) had owned a dog, cat, and another animal, and 67 (1.4%) had owned a cat and another animal. Table 1 shows participant sociodemographic and comprehensive health characteristics in relation to dog/cat ownership status. As compared with never owners, current and past dog/cat owners were more likely to be women (P < .001), to be young (P < .001), and to have a higher household size (P < .001), higher education level (P < .001), higher equivalent income (P < .001), and higher TMIG-IC score (P < .001). They were also more likely to have received a physician diagnosis of cancer (P = .003), to have been hospitalized during the past year (P < .001), to have experienced a fall during the past year (P = .005), and to drink alcohol and smoke (P < .001) and were less likely to live alone (P < .001). In addition, they had higher motor fitness scale scores (P < .001)and reported greater walking activity (P = .007), in the assessment of physical function and physical activity. For social function, current and past dog/cat owners had greater interaction with neighbors (P < .001) and higher trust in neighbors (P < .001) and were less likely to be socially isolated (P < .001). Analysis of psychological function showed that they had higher subjective happiness (P < .001), better self-rated health (P < .001), a higher WHO-5 score (P < .001), and a lower GDS-5 score (P = .002).

**Table 1:** Associations of sociodemographic and comprehensive health characteristics with current and past dog/cat ownership among community-dwelling older Japanese.

Variable	Dog or Cat ownership			
	Current (n = 1545; 13.8%)	Past (n = 3311; 29.5%)	Never (n = 6377; 56.8%)	P-Value
**DEMOGRAPHICS and CHARACTERISTICS**				
Sex (female)	52.7	53.3	50.4	< .001
Age, years (%)				< .001
65–74	59.4	46.3	45.6	
75–84	40.6	53.7	54.4	
Living alone (%)	9.3	18.9	24.3	< .001
Household size	2.7 (1.2)	2.3 (1.1)	2.2 (1.1)	< .001
Marital status (%)				< .001
Married	74.0	68.6	64.7	
Divorced	5.7	5.7	6.4	
Widowed	17.4	20.4	19.7	
Single	3.0	5.3	9.3	
Educational attainment (%)				< .001
Elementary school	0.9	1.3	1.7	
Middle school	18.2	18.9	26.7	
High school	37.0	37.1	39.7	
College, university, or graduate school	41.8	40.9	30.0	
Others	2.2	1.8	1.9	
Equivalent income (%)				< .001
<1,000,000 yen	6.7	5.6	6.2	
1,000,000 yen—2,500,000 yen	32.6	33.9	39.7	
2,500,000 yen—4,000,000 yen	18.8	21.7	21.0	
≥4,000,000 yen	21.4	19.2	13.5	
Unknown	20.5	19.5	19.5	
Employment (%)				< .001
Presence	34.7	29.2	25.6	
Chronic disease (%)				
Hypertension	54.2	54.0	54.6	.615
Hyperlipidemia	44.6	42.1	41.9	.276
Heart disease	21.2	22.9	21.7	.149
Stroke	8.5	7.8	7.5	.084
Diabetes mellitus	18.8	18.5	18.8	.864
Bone and joint disease	30.4	32.2	31.9	.849
Lung respiratory disease	14.3	16.4	14.6	.103
Cancer	18.2	17.2	16.1	.003
Chronic pain (%)				
Shoulder	14.1	13.3	12.8	.258
Waist	24.5	24.4	23.2	.055
Knee	19.4	18.8	20.1	.373
Hospitalization during the past year (%)	14.0	13.1	12.2	< .001
Fall during the past year (%)	15.2	16.5	14.2	.005
Alcohol drinking status (%)				< .001
Current	57.2	56.0	54.0	
Past	6.7	8.6	8.3	
Never	36.1	35.4	37.7	
Smoking status (%)				< .001
Current	14.6	11.9	12.5	
Past	34.0	34.6	31.6	
Never	51.3	53.5	55.9	
Sleep duration (min)	397.0 (71.3)	395.3 (72.5)	396.7 (74.2)	.446
Food variety (score)	3.1 (2.2)	3.2 (2.2)	3.2 (2.2)	.590
TMIG-IC (score)	11.5 (1.9)	11.6 (1.8)	11.3 (2.0)	< .001
**PHYSICAL FUNCTION, PHYSICAL ACTIVITY**				
Mobility limitation (%)	26.8	28.7	30.5	.157
BMI (kg/m^2^)	22.9 (3.3)	22.6 (3.2)	22.7 (3.2)	.625
Motor fitness scale (score)	10.9 (3.2)	10.8 (3.2)	10.4 (3.3)	< .001
Physical activity (MET-hours/week)				
Vigorous physical activity	14.1 (32.1)	15.5 (34.3)	14.7 (33.3)	.876
Moderate physical activity	8.5 (18.5)	8.3 (18.3)	7.9 (17.9)	.320
Walking activity	25.4 (24.6)	23.2 (23.7)	23.1 (22.9)	.007
Moderate to vigorous physical activity	44.7 (54.8)	44.3 (55.8)	43.2 (54.8)	.479
Frailty (%)	22.3	23.4	24.7	.063
**SOCIAL FUNCTION**				
Interaction with neighbors (%)				< .001
Significant relationship	24.2	25.2	22.0	
Conversation	40.2	38.4	36.3	
Exchange of greetings only	30.8	30.9	33.9	
No social contact	4.7	5.6	7.8	
Social isolation (yes %)	23.3	24.9	36.3	< .001
Trust in neighbors (yes %)	80.7	79.3	75.1	< .001
Frequency of going outdoors (%)				.491
At least once a day	79.0	72.9	74.4	
Once every 2–3 days	14.6	20.0	18.2	
Less than once a week	6.4	7.1	7.4	
**PSYCHOLOGICAL FUNCTION**				
Subjective happiness: happy, rather happy (%)	95.4	94.4	92.9	< .001
Self-rated health (%)				< .001
Excellent to good	83.4	82.4	79.9	
Fair to poor	16.6	17.6	20.1	
GDS-5 (score)	1.2 (1.3)	1.3 (1.3)	1.3 (1.3)	.002
WHO-5 (score)	62.5 (23.3)	62.2 (23.3)	60.2 (24.4)	< .001

(SD). P values were calculated by using a cumulative logit model (adjusted for sex and age)

BMI, body mass index. TMIG-IC, Tokyo Metropolitan Institute of Gerontology Index of Competence. GDS, Geriatric Depression Scale.

Table 2 shows independent associations of health characteristics with dog/cat ownership, after adjustment for important sociodemographic and health characteristics. In the analysis of physical function and physical activity, the odds ratio (OR) for a 1-point increase in the motor fitness scale score among current and past dog/cat owners was 1.01 (95% confidence interval: 1.01–1.02), and the OR for a 10-MET-hour/week increase in walking activity was 1.02 (1.01–1.04). In the analysis of social function, the ORs for interaction with neighbors among current and past dog/cat owners were 1.27 (1.06–1.52) for exchange of greetings only, 1.49 (1.24–1.79) for conversation-level interaction, and 1.64 (1.35–2.00) for a close relationship, respectively, as compared with participants reporting no social contact. As compared with participants without social isolation, the OR for social isolation was 0.74 (0.66–0.80). The OR for trust in neighbors was 1.24 (1.12–1.38) as compared with participants without trust in neighbors. In contrast, there was no significant association between psychological function and dog/cat ownership after controlling for important sociodemographic and health characteristics.

**Table 2:** Independent associations of health xharacteristics with xurrent and past dog/xat ownership among community-dwelling older Japanese.

Independent Variable	Odds Ratio (95% Confidence Interval)
**PHYSICAL FUNCTION, PHYSICAL ACTIVITY**	
Motor fitness scale (per 1-point increase)	1.01 (1.01–1.02) *
Walking activity (per 10-MET-hours/week increase)	1.02 (1.01–1.04) *
**SOCIAL FUNCTION**	
Interaction with neighbors: No social contact§	1
Exchange of greetings only	1.27 (1.06–1.52) *
Conversation	1.49 (1.24–1.79) **
Significant relationship	1.64 (1.35–2.00) **
Social isolation: no§	1
yes	0.74 (0.66–0.80) **
Trust in neighbors: no §	1
yes	1.24 (1.12–1.38) **
**PSYCHOLOGICAL FUNCTION**	
Subjective happiness: rather unhappy, unhappy,§	1
happy, rather happy	1.09 (0.91–1.31)
Self-rated health: Fair to poor§	1
Excellent to good	1.07 (0.95–1.20)
GDS-5	1.01 (0.98–1.05)
WHO-5 (per 10-point increase)	1.01 (0.99–1.03)

*P < .05.

**P < .01.

§ reference group.

Mixed-effects cumulative logistic regression models were run separately. The random effects were the 18 administrative districts.

Analysis adjusted for sex, age, household size, educational attainment, equivalent income, history of cancer, hospitalization during the past year, fall during the past year, alcohol drinking status, and TMIG-IC score.

In addition, we analyzed associations of health characteristics with dog ownership and cat ownership, separately. Among health characteristics, motor fitness scale, walking activity, interaction with neighbors, social isolation, and trust in neighbors showed significant associations with dog ownership, after adjustment for important confounders. After adjustment for important confounders, cat ownership was significantly associated with interaction with neighbors, social isolation, and trust in neighbors but not with motor fitness scale or walking (Tables A, B, and C in S1 File).

## Discussion

Although, we hypothesized that that dog/cat owners have higher social function and/or psychological function, in addition to higher physical function and physical activity, physical function and physical activity and social function showed significant association with dog or cat ownership in our comprehensive analysis among community-dwelling older Japanese. Owen et al. reported that children from dog-owning families spent more time in light or moderate-to-vigorous physical activity than did children without dogs [4]. Dall et al. reported that older dog owners spent 20 minutes longer walking than did non–dog owners [13]. Although our results from community-dwelling older Japanese showed no significant association of moderate-to-vigorous physical activity with dog or cat ownership, walking activity was greater for current and past dog/cat owners than for never owners, even after adjustment for important sociodemographic and health characteristics. This association was stronger for dog owners. These results suggest that dog ownership is associated with more light physical activity, over a wide age range, among family members. Moreover, dog walking increases total walking time for older persons and helps maintain their motor fitness.

Higher social function, including better interaction with neighbors, less social isolation, and more trust in neighbors showed significant association with current and past dog/cat owners. These associations were observed for both dog and cat owners. Nagasawa et al. reported that dog ownership at an early age significantly increased scores for the companionship factor and social support factor in later life [11]. Wood et al. suggested that pets affect broader social interactions and perceptions, sense of community, and social capital at the neighborhood level [28]. Causality cannot be established in this cross-sectional study; however, the results suggest that dog/cat owners have increased social function because of their greater opportunity to participate in pet-related social activities.

Sex- and age-adjusted univariate cumulative logit models showed significant associations between some psychological factors and dog/cat ownership; however, after adjustment for potential confounders, mixed-effects cumulative logistic regression models showed no significant associations with dog or cat ownership. Turner et al. reported that cat ownership was related to fewer episodes of bad mood [29]. In addition, non–pet owners felt lonely twice as frequently as pet owners [28]. Although their studies did not include important sociodemographic and characteristics, it is plausible that pet ownership has psychological effects. Future studies should explore independent associations of other measures of psychological function with dog/cat ownership.

This study has strengths that warrant mention. First, the analysis of sociodemographic and health characteristics was comprehensive, which enabled us to examine independent associations of physical function, physical activity, social function, and psychological function with dog/cat ownership, after controlling for important sociodemographic and health characteristics. Second, our large sample of community-dwelling older Japanese enabled subgroup analysis of current, past, and never ownership of dogs and cats. Moreover, we were able to examine dog and cat ownership separately.

This study has some limitations. First, although we collected information on dog and cat ownership, data on years of ownership and frequency of dog walking were not available for analysis. Second, the population of dog/cat owners is smaller than those in Western countries [30–32]. The Japan Pet Food Association reported that only 15% of Japanese households have a dog and 10% have a cat. Cultures might differ in their relationships to pet animals, and future studies should examine associations of health characteristics with dog/cat ownership among Western populations. Third, although the present cross-sectional study showed independent associations of health characteristics with dog/cat ownership after adjustment for important sociodemographic and characteristics, causality cannot be established. A well-designed study will need to explore longitudinal effects of dog/cat ownership on health outcomes in later life. Finally, companion animal ownership has disadvantages (i.e. zoonosis, animal allergy, and pet loss) and imposes limitations on owners (i.e. fewer residential choices and cost). Future studies should examine the advantages of pet ownership after considering its disadvantages and relevant social and environmental factors.

To our knowledge, this is the first study to identify sociodemographic characteristics in a large sample of community-dwelling older dog/cat owners. This cross-sectional study of a large sample of community-dwelling older adults showed that dog owners have greater motor fitness and walk more and that dog and cat owners have higher social function than never owners, even after adjustment for important sociodemographic and health characteristics. Dog walking might increase walking time for older persons and help maintain motor fitness, regardless of family support or financial resources. In addition, dog and cat owners may have more opportunities to participate in social activities. Caring for a dog or cat might be an effective health promotion strategie to increase physical activity and facilitate social participation among older adults.

## Supporting information

S1 File (A) Associations of Sociodemographic and Health Characteristics with Current/Past Dog Ownership Among Community-Dwelling Older Japanese. (B) Associations of Sociodemographic and Health Characteristics with Current/Past Cat Ownership Among Community-Dwelling Older Japanese. (C) Independent Associations of Health Characteristics with Current and Past Dog/Cat Ownership Among Community-Dwelling Older Japanese.
(DOCX)

S2 File Questionnaire. (DOCX)

## Abbreviations

TMIG-IC: Tokyo Metropolitan Institute of Gerontology Index of Competence
GDS: Geriatric Depression Scale
WHO: World Health Organization Five
