# Correction: Physical, social, and psychological characteristics of community-dwelling elderly Japanese dog and cat owners

**DOI:** 10.1371/journal.pone.0214824

**Published:** 2019-03-28

**Authors:** Yu Taniguchi, Satoshi Seino, Mariko Nishi, Yui Tomine, Izumi Tanaka, Yuri Yokoyama, Hidenori Amano, Akihiko Kitamura, Shoji Shinkai

## Notice of republication

This article was republished on March 20, 2019 to remove a Supporting Information file that was incorrectly included in the originally published article. The article’s Data Availability statement has also been updated to reflect this change. Please download this article again to view the correct version.
