# Correction: Physical, social, and psychological characteristics of community-dwelling elderly Japanese dog and cat owners

**DOI:** 10.1371/journal.pone.0229163

**Published:** 2020-02-10

**Authors:** The PLOS ONE Staff

In the title of Table 2, the words “characteristics,” “current,” and “cat” are misspelled. The correct title is: Table 2. Independent associations of health characteristics with current and past dog/cat ownership among community-dwelling older Japanese. The publisher apologizes for the error.

**Table 2:** Independent associations of health characteristics with current and past dog/cat ownership among community-dwelling older Japanese.

Independent Variable	Odds Ratio (95% Confidence Interval)
PHYSICAL FUCNTION, PHYSICAL ACTIVITY	
Motor fitness scale (per 1-point increase)	1.01 (1.01–1.02) *
Walking activity (per 10-MET-hours/week increase)	1.02 (1.01–1.04) *
**SOCIAL FUNCTION**	
Interaction with neighbors: No social contact§	1
Exchange of greetings only	1.27 (1.06–1.52) *
Conversation	1.49 (1.24–1.79) **
Significant relationship	1.64 (1.35–2.00) **
Social isolation: no§	1
yes	0.74 (0.66–0.80) **
Trust in neighbors: no §	1
yes	1.24 (1.12–1.38) **
**PSYCHOLOGICAL FUNCTION**	
Subjective happiness: rather unhappy, unhappy, §	1
happy, rather happy	1.09 (0.91–1.31)
Self-rated health: Fair to poor§	1
Excellent to good	1.07 (0.95–1.20)
GDS-5	1.01 (0.98–1.05)
WHO-5 (per 10-point increase)	1.01 (0.99–1.03)

*P < .05,

**P < .01;

§ reference group.

Mixed-effects cumulative logistic regression models were run separately. The random effects were the 18 administrative districts.

Analysis adjusted for sex, age, household size, educational attainment, equivalent income, history of cancer, hospitalization during the past year, fall during the past year, alcohol drinking status, and TMIG-IC score.
