# Supplementary material for: Physical, social, and psychological characteristics of community-dwelling elderly Japanese dog and cat owners
Source: PLoS One. 2018 Nov 14;13(11):e0206399. doi: 10.1371/journal.pone.0206399 (PMC6241120; doi:10.1371/journal.pone.0206399)
Supplement: S1 File — (A) Associations of Sociodemographic and Health Characteristics with Current/Past Dog Ownership Among Community-Dwelling Older Japanese. (B) Associations of Sociodemographic and Health Characteristics with Current/Past Cat Ownership Among Community-Dwelling Older Japanese. (C) Independent Associations of Health Characteristics with Current and Past Dog/Cat Ownership Among Community-Dwelling Older Japanese. (DOCX) [file pone.0206399.s001.docx]

**S1 table A. Associations of Sociodemographic and Health Characteristics with Current/Past Dog Ownership Among Community-Dwelling Older Japanese.**

| Variable | Dog ownership | | | |
| --- | --- | --- | --- | --- |
|  | Current (n=963; 8.6%) | Past (n=2540; 22.6%) | Never (n=7730; 68.8%) | P-Value |
| **DEMOGRAPHICS and CHARACTERISTICS** |  |  |  |  |
| Sex (female) | 54.4 | 52.4 | 50.9 | .003 |
|  |  |  |  |  |
| Age, years (%) |  |  |  | <.001 |
| 65-74 | 59.0 | 46.8 | 46.6 |  |
| 75-84 | 41.0 | 53.2 | 53.4 |  |
|  |  |  |  |  |
| Living alone (%) | 8.6 | 17.9 | 23.1 | <.001 |
|  |  |  |  |  |
| Household size | 2.7 (1.2) | 2.4 (1.2) | 2.2 (1.1) | <.001 |
|  |  |  |  |  |
| Marital status (%) |  |  |  | <.001 |
| Married | 74.0 | 69.9 | 65.3 |  |
| Divorced | 5.8 | 5.6 | 6.3 |  |
| Widowed | 18.1 | 19.7 | 19.7 |  |
| Single | 2.1 | 4.9 | 8.6 |  |
|  |  |  |  |  |
| Educational attainment (%) |  |  |  | <.001 |
| Elementary school | 0.8 | 1.1 | 1.7 |  |
| Middle school | 17.2 | 17.9 | 25.8 |  |
| High school | 37.8 | 36.9 | 39.2 |  |
| College, university, or graduate school | 42.0 | 42.4 | 31.5 |  |
| Other | 2.2 | 1.7 | 1.9 |  |
|  |  |  |  |  |
| Equivalent income (%) |  |  |  | <.001 |
| <1,000,000 yen | 7.3 | 5.8 | 6.1 |  |
| 1,000,000 yen - 2,500,000 yen | 31.4 | 32.2 | 39.3 |  |
| 2,500,000 yen - 4,000,000 yen | 17.4 | 21.8 | 21.1 |  |
| ≥4,000,000 yen | 22.5 | 20.0 | 14.2 |  |
| Unknown | 21.4 | 20.2 | 19.3 |  |
|  |  |  |  |  |
| Employment (%) |  |  |  | <.001 |
| Presence | 35.4 | 29.4 | 26.5 |  |
|  |  |  |  |  |
| Chronic disease (%) |  |  |  |  |
| Hypertension | 53.3 | 53.9 | 54.4 | .518 |
| Hyperlipidemia | 44.5 | 42.5 | 41.9 | .320 |
| Heart disease | 21.5 | 22.7 | 21.8 | .239 |
| Stroke | 8.4 | 7.7 | 7.7 | .405 |
| Diabetes mellitus | 18.5 | 18.3 | 18.9 | .811 |
| Bone and joint disease | 30.1 | 32.0 | 31.9 | .678 |
| Lung respiratory disease | 13.8 | 17.1 | 14.6 | .044 |
| Cancer | 18.0 | 17.8 | 16.2 | .007 |
|  |  |  |  |  |
| Chronic pain (%) |  |  |  |  |
| Shoulder | 13.7 | 13.8 | 12.8 | .244 |
| Waist | 23.5 | 24.7 | 23.5 | .278 |
| Knee | 20.0 | 18.0 | 20.1 | .148 |
|  |  |  |  |  |
| Hospitalization during the past year (%) | 14.8 | 13.3 | 12.2 | .004 |
|  |  |  |  |  |
| Fall during the past year (%) | 15.3 | 16.3 | 14.5 | .028 |
|  |  |  |  |  |
| Alcohol drinking status (%) |  |  |  | .009 |
| Current | 57.4 | 56.4 | 54.3 |  |
| Past | 5.8 | 8.6 | 8.4 |  |
| Never | 36.7 | 35.0 | 37.4 |  |
|  |  |  |  |  |
| Smoking status (%) |  |  |  | .008 |
| Current | 13.9 | 11.7 | 12.7 |  |
| Past | 33.9 | 35.2 | 31.9 |  |
| Never | 52.2 | 53.1 | 55.4 |  |
|  |  |  |  |  |
| Sleep duration (min) | 396.9 (68.8) | 396.2 (72.9) | 396.3 (74.0) | .358 |
|  |  |  |  |  |
| Food variety (score) | 3.0 (2.2) | 3.3 (2.2) | 3.2 (2.2) | .329 |
|  |  |  |  |  |
| TMIG-IC (score) | 11.6 (1.8) | 11.6 (1.9) | 11.3 (1.9) | <.001 |
|  |  |  |  |  |
| **PHYSICAL FUNCTION, PHYSICAL ACTIVITY** |  |  |  |  |
| Mobility limitation (%) | 25.5 | 27.6 | 30.5 | .002 |
|  |  |  |  |  |
| BMI (kg/m^2^) | 23.0 (3.3) | 22.6 (3.2) | 22.7 (3.2) | .966 |
|  |  |  |  |  |
| Motor fitness scale (score) | 11.0 (3.1) | 10.9 (3.2) | 10.5 (3.3) | <.001 |
|  |  |  |  |  |
| Physical activity |  |  |  |  |
| Vigorous physical activity (MET-hours/week) | 14.5 (33.0) | 15.3 (33.3) | 14.8 (33.5) | .783 |
| Moderate physical activity (MET-hours/week) | 8.0 (18.1) | 8.4 (18.3) | 8.0 (18.2) | .740 |
| Walking activity (MET-hours/week) | 26.9 (24.7) | 23.1 (23.5) | 23.2 (23.2) | .034 |
| Moderate to vigorous physical activity (MET-hours/week) | 46.0 (54.0) | 44.3 (55.5) | 43.3 (55.1) | .292 |
|  |  |  |  |  |
| Frailty (%) | 21.0 | 22.0 | 24.6 | .008 |
|  |  |  |  |  |
| **SOCIAL FUNCTION** |  |  |  |  |
| Interaction with neighbors (%) |  |  |  | <.001 |
| Significant relationship | 25.5 | 24.6 | 22.5 |  |
| Conversation | 40.6 | 38.3 | 36.7 |  |
| Exchange of greetings only | 29.7 | 33.6 | 33.3 |  |
| No social contact | 4.2 | 5.4 | 7.5 |  |
|  |  |  |  |  |
| Social isolation, yes (%) | 21.1 | 24.8 | 31.0 | <.001 |
|  |  |  |  |  |
| Trust in neighbors, yes (%) | 81.1 | 79.0 | 76.0 | <.001 |
|  |  |  |  |  |
| Frequency of going outdoors (%) |  |  |  | .118 |
| At least once a day | 81.0 | 73.1 | 74.3 |  |
| Once every 2-3 days | 14.7 | 19.5 | 18.3 |  |
| Less than once a week | 4.3 | 7.5 | 7.4 |  |
|  |  |  |  |  |
| **PSYCHOLOGICAL FUNCTION** |  |  |  |  |
| Subjective happiness: happy, rather happy (%) | 95.0 | 95.0 | 93.1 | <.001 |
|  |  |  |  |  |
| Self-rated health (%) |  |  |  | .002 |
| Excellent to good | 83.1 | 83.1 | 80.2 |  |
| Fair to poor | 16.9 | 16.9 | 19.8 |  |
|  |  |  |  |  |
| GDS-5 (score) | 1.2 (1.2) | 1.3 (1.3) | 1.3 (1.3) | .018 |
|  |  |  |  |  |
| WHO-5 (score) | 63.4 (23.0) | 62.7 (23.2) | 60.2 (24.3) | <.001 |
|  |  |  |  |  |

(SD). P-values were calculated with a cumulative logit model (adjusted for sex and age)

BMI, body mass index. TMIG-IC, Tokyo Metropolitan Institute of Gerontology Index of Competence. GDS, Geriatric Depression Scale.

**S2 table B. Associations of Sociodemographic and Health Characteristics with Current/Past Cat Ownership Among Community-Dwelling Older Japanese.**

| Variable | Cat ownership | | | |
| --- | --- | --- | --- | --- |
|  | Current (n=706; 6.3%) | Past (n=1242; 11.1%) | Never (n=9285; 82.6%) | P-Value |
| **DEMOGRAPHICS and CHARACTERISTICS** |  |  |  |  |
| Sex (female) | 52.1 | 55.0 | 51.0 | .003 |
|  |  |  |  |  |
| Age, years (%) |  |  |  | <.001 |
| 65-74 | 60.5 | 45.5 | 47.1 |  |
| 75-84 | 39.5 | 54.5 | 52.9 |  |
|  |  |  |  |  |
| Living alone (%) | 10.5 | 22.3 | 21.2 | <.001 |
|  |  |  |  |  |
| Household size | 2.6 (1.2) | 2.2 (1.1) | 2.3 (1.1) | <.001 |
|  |  |  |  |  |
| Marital status (%) |  |  |  | <.001 |
| Married | 74.0 | 63.9 | 67.0 |  |
| Divorced | 5.6 | 6.9 | 6.0 |  |
| Widowed | 16.2 | 22.0 | 19.5 |  |
| Single | 4.2 | 7.2 | 7.5 |  |
|  |  |  |  |  |
| Education attainment (%) |  |  |  | <.001 |
| Elementary school | 1.2 | 1.2 | 1.5 |  |
| Middle school | 19.9 | 19.6 | 24.0 |  |
| High school | 35.3 | 37.2 | 38.9 |  |
| College, university, or graduate school | 41.6 | 39.9 | 33.7 |  |
| Others | 2.0 | 2.0 | 1.9 |  |
|  |  |  |  |  |
| Equivalent income (%) |  |  |  | .011 |
| <1,000,000 yen | 5.9 | 5.3 | 6.2 |  |
| 1,000,000 yen - 2,500,000 yen | 34.0 | 36.2 | 37.4 |  |
| 2,500,000 yen - 4,000,000 yen | 20.4 | 22.5 | 20.8 |  |
| ≥4,000,000 yen | 20.0 | 17.4 | 15.8 |  |
| Unknown | 19.7 | 18.6 | 19.8 |  |
|  |  |  |  |  |
| Employment (%) |  |  |  | <.001 |
| Presence | 36.0 | 29.1 | 27.2 |  |
|  |  |  |  |  |
| Chronic disease (%) |  |  |  |  |
| Hypertension | 51.2 | 53.0 | 54.8 | .151 |
| Hyperlipidemia | 44.1 | 42.3 | 42.2 | .639 |
| Heart disease | 20.5 | 23.4 | 21.9 | .399 |
| Stroke | 9.3 | 7.7 | 7.6 | .129 |
| Diabetes mellitus | 18.1 | 19.7 | 18.6 | .481 |
| Bone and joint disease | 30.6 | 33.8 | 31.6 | .409 |
| Lung respiratory disease | 14.8 | 17.4 | 14.8 | .051 |
| Cancer | 17.7 | 17.2 | 16.6 | .176 |
|  |  |  |  |  |
| Chronic pain (%) |  |  |  |  |
| Shoulder | 14.4 | 12.7 | 13.1 | .793 |
| Waist | 24.8 | 24.0 | 23.6 | .389 |
| Knee | 18.7 | 20.7 | 19.6 | .693 |
|  |  |  |  |  |
| Hospitalization during the past year (%) | 12.4 | 12.4 | 12.7 | .989 |
|  |  |  |  |  |
| Fall during the past year (%) | 14.8 | 17.9 | 14.6 | .017 |
|  |  |  |  |  |
| Alcohol drinking status (%) |  |  |  | .309 |
| Current | 55.7 | 54.6 | 55.0 |  |
| Past | 8.5 | 9.8 | 8.0 |  |
| Never | 35.8 | 35.5 | 37.0 |  |
|  |  |  |  |  |
| Smoking status (%) |  |  |  | <.001 |
| Current | 16.2 | 12.1 | 12.4 |  |
| Past | 33.2 | 34.5 | 32.6 |  |
| Never | 50.6 | 53.6 | 55.1 |  |
|  |  |  |  |  |
| Sleep duration (min) | 396.8 (73.9 | 393.4 (71.8 | 396.7 (73.5 | .720 |
|  |  |  |  |  |
| Food variety (score) | 3.2 (2.2 | 3.2 (2.2 | 3.2 (2.2 | .312 |
|  |  |  |  |  |
| TMIG-IC (score) | 11.4 (2.0 | 11.6 (1.8 | 11.4 (1.9 | .007 |
|  |  |  |  |  |
| **PHYSICAL FUNCTION, PHYSICAL ACTIVITY** |  |  |  |  |
| Mobility limitation (%) | 28.3 | 30.4 | 29.4 | .371 |
|  |  |  |  |  |
| BMI (kg/m^2^) | 22.7 (3.3) | 22.7 (3.1) | 22.7 (3.2) | .803 |
|  |  |  |  |  |
| Motor fitness scale (score) | 10.8 (3.2) | 10.6 (3.2) | 10.6 (3.3) | .872 |
|  |  |  |  |  |
| Physical activity |  |  |  |  |
| Vigorous physical activity (MET-hours/week) | 14.0 (32.0) | 16.4 (37.0) | 14.7 (33.0) | .450 |
| Moderate physical activity (MET-hours/week) | 8.6 (18.1) | 8.8 (19.1) | 8.0 (17.9) | .214 |
| Walking activity (MET-hours/week) | 23.7 (24.1) | 24.3 (24.0) | 23.3 (23.3) | .400 |
| Moderate to vigorous physical activity (MET-hours/week) | 42.9 (55.0) | 45.4 (56.8) | 43.6 (54.8) | .736 |
|  |  |  |  |  |
| Frailty (%) | 23.4 | 22.8 | 23.8 | .906 |
|  |  |  |  |  |
| **SOCIAL FUNCTION** |  |  |  |  |
| Interaction with neighbors (%) |  |  |  | <.001 |
| Significant relationship | 23.5 | 26.3 | 22.8 |  |
| Conversation | 40.4 | 38.6 | 37.1 |  |
| Exchange of greetings only | 30.9 | 29.1 | 33.2 |  |
| No social contact | 5.1 | 6.1 | 6.9 |  |
|  |  |  |  |  |
| Social isolation, yes (%) | 25.8 | 25.1 | 29.5 | .002 |
|  |  |  |  |  |
| Trust in neighbors, yes (%) | 81.1 | 78.4 | 76.7 | .005 |
|  |  |  |  |  |
| Frequency of going outdoors (%) |  |  |  | .700 |
| At least once a day, | 76.7 | 73.9 | 74.5 |  |
| Once every 2-3 days | 14.0 | 20.0 | 18.3 |  |
| Less than once a week | 9.3 | 6.1 | 7.2 |  |
|  |  |  |  |  |
| **PSYCHOLOGICAL FUNCTION** |  |  |  |  |
| Subjective happiness: happy, rather happy (%) | 96.1 | 93.5 | 93.5 | .100 |
|  |  |  |  |  |
| Self-rated health (%) |  |  |  | .414 |
| Excellent to good | 83.6 | 80.9 | 80.9 |  |
| Fair to poor | 16.4 | 19.1 | 19.1 |  |
|  |  |  |  |  |
| GDS-5 (score) | 1.3 (1.3) | 1.3 (1.3) | 1.3 (1.3) | .892 |
|  |  |  |  |  |
| WHO-5 (score) | 61.4 (23.6) | 60.9 (23.4) | 61.1 (24.1) | .961 |
|  |  |  |  |  |

(SD). P-values were calculated with a cumulative logit model (adjusted for sex and age)

BMI, body mass index. TMIG-IC, Tokyo Metropolitan Institute of Gerontology Index of Competence. GDS, Geriatric Depression Scale.

**S3 table C. Independent Associations of Health Characteristics with Current and Past Dog/Cat Ownership Among Community-Dwelling Older Japanese.**

|  | Dog owners | Cat owners |
| --- | --- | --- |
| Independent Variable | Odds Ratio (95% Confidence Interval) | Odds Ratio (95% Confidence Interval) |
| **PHYSICAL FUNCTION, PHYSICAL ACTIVITY** |  |  |
| Mobility limitation: no § | 1 | - |
| yes | 0.94 (0.84-1.05) | - |
| Motor fitness scale (per 1-point increase) | 1.03 (1.01-1.04) * | - |
| Walking activity (per 10-MET-hours/week increase) | 1.02 (1.01-1.04) * | - |
| Frailty: no § | 1 | - |
| yes | 1.06 (0.94-1.20) | - |
| **SOCIAL FUNCTION** |  |  |
| Interaction with neighbors: No social contact § | 1 | 1 |
| Exchange of greetings only | 1.30 (1.06-1.61) ** | 1.07 (0.85-1.36) |
| Conversation | 1.47 (1.19-1.82) ** | 1.26 (10.99-1.60) |
| Significant relationship | 1.62 (1.30-2.02) ** | 1.32 (1.03-1.70) * |
| Social isolation: no§ | 1 | 1 |
| yes | 0.75 (0.67-0.84) ** | 0.85 (0.75-0.96) * |
| Trust in neighbors: no § | 1 | 1 |
| yes | 1.15 (1.03-1.29) * | 1.15 (1.01-1.31) * |
| **PSYCHOLOGICAL FUNCTION** |  |  |
| Subjective happiness: rather unhappy, unhappy,§ | 1 | - |
| happy, rather happy | 1.05 (0.86-1.29) | - |
| Self-rated health: Fair to poor§ | 1 | - |
| Excellent to good | 1.03 (0.91-1.18) | - |
| GDS-5 | 1.02 (0.98-1.06) | - |
| WHO-5 (per 10-point increase) | 1.02 (1.00-1.04) | - |

*P<.05, **P<.01; OR, odds ratio; CI, confidence interval; § reference group. Mixed-effects cumulative logistic regression models were run separately. The random effects were the 18 administrative districts.

Analysis of dog owners was adjusted for sex, age, household size, educational attainment, equivalent income, history of lung respiratory disease, history of cancer, hospitalization during the past year, fall during the past year, alcohol drinking status, and TMIG-IC score.

Analysis of cat owners was adjusted for sex, age, household size, educational attainment, equivalent income, fall during the past year, and TMIG-IC score.
