# Supplementary material for: Physical, social, and psychological characteristics of community-dwelling elderly Japanese dog and cat owners
Source: PLoS One. 2018 Nov 14;13(11):e0206399. doi: 10.1371/journal.pone.0206399 (PMC6241120; doi:10.1371/journal.pone.0206399)
Supplement: S2 File — (DOCX) [file pone.0206399.s002.docx]

QUESTIONNAIRE

Questions1. Have you ever had a pet?

Responses1. Current, Past, Never

Questions2 (For the Current or past owener). What kind of ped have you had (or did you have)?

Responses2. Dog, Cat, The others

質問項目

質問1. あたなはペットを飼ったことがありますか？

回答1. 現在飼っている、以前飼っていたが現在飼っていない、飼ったことがない

質問2 (ペット飼育経験者のみ). どんなペットを飼っていますか（または飼っていましたか）？

回答2. 犬、猫、その他
